# Acid Resistance of CAD/CAM Resin Composites

**DOI:** 10.3390/biomedicines10061383

**Published:** 2022-06-11

**Authors:** Leonie Schmohl, Anuschka Josephine Roesner, Florian Fuchs, Maximilian Wagner, Michael Benno Schmidt, Sebastian Hahnel, Angelika Rauch, Andreas Koenig

**Affiliations:** 1 Department of Prosthetic Dentistry and Dental Material Science, Leipzig University, Liebigstraße 12, 04103 Leipzig, Germany; florian.fuchs@medizin.uni-leipzig.de (F.F.); maximilian.wagner@iom-leipzig.de (M.W.); michael3.schmidt@klinik.uni-regensburg.de (M.B.S.); sebastian.hahnel@klinik.uni-regensburg.de (S.H.); angelika.rauch@klinik.uni-regensburg.de (A.R.); akoenig@uni-leipzig.de (A.K.); 2 Department of Prosthetic Dentistry, Faculty of Medicine, Medical Center, Center for Dental Medicine University of Freiburg, Hugstetter Straße 55, 79106 Freiburg, Germany; anuschka.roesner@uniklinik-freiburg.de; 3 Department of Functional Surfaces, Leibniz Institute for Surface Engineering, Permoserstraße 15, 04318 Leipzig, Germany; 4 Department of Prosthetic Dentistry, UKR University Hospital Regensburg, Franz-Josef-Strauß-Allee 11, 93053 Regensburg, Germany

**Keywords:** resin-based composites (RBC), erosion-related material surface, soft drink, computer-aided design, computer-aided manufacturing

## Abstract

Acid resistance of CAD/CAM resin composites. Erosion-related tooth surface loss is closely related to acid exposure, such as contact with acidic beverages or disease-related reflux. As a result, dental restorations in affected patients are also exposed to acids, which indicates that the performance and longevity of a dental restoration is impacted by the acid resistance of the individually employed restorative materials. However, unlike for ceramic materials, the acid resistance of CAD/CAM resin composites is not commonly evaluated by the manufacturers, and no standardised test methods have yet been established. Against this background, the present in vitro study aimed to examine the long-term resistance of CAD/CAM resin composites (Brilliant Crios, Cerasmart, Grandio blocs, Lava Ultimate, Shofu Block HC) against three acidic media (tonic water, acetic acid, hydrochloric acid) as well as demineralized water and to investigate potential damage mechanisms. Changes in surface roughness (Sa) were detected by confocal laser scanning microscopy (CLSM), and changes in surface hardness were measured using Vickers hardness (HV). The damage mechanisms were analysed by scanning electron microscopy (SEM) with energy dispersive X-ray spectroscopy (EDS) and micro X-ray computer tomography (µXCT). For each material, few changes in either Sa or HV were identified for at least one of the different media; for Cerasmart, the sharpest deterioration in surface properties was observed. SEM–EDS revealed leaching of barium, aluminium, and titanium from fillers in a 2 µm zone on the rough but not on the polished surface of the specimen. Within the limitations of the current study, it can be concluded that polished CAD/CAM resin composites can be recommended for clinical use in patients with erosive conditions.

## 1. Introduction

Aetiologically, tooth surface loss is the sum of cumulative, multifactorial events, which ultimately lead to an irreversible loss of superficial tooth structure. In addition to idiopathic and genetic factors, mechanical or chemical processes can also cause tooth surface loss [1]. Whereas attrition describes intrinsic mechanical wear, which is caused by direct functional or parafunctional tooth-to-antagonist contact [1], abrasion refers to extrinsic mechanical wear. The latter is not related to the function or parafunctions of the masticatory system, but rather to oral hygiene measures, such as excessive occlusal contact pressure during tooth brushing or to habits such as fingernail or pin chewing [2,3]. Chemical tooth surface loss is closely connected with erosion, which is defined as a pathological, noncariogenic destruction process of the tooth surface caused by the action of acids without obligatory bacterial involvement [2,4]. Aetiologically, exogenous (extrinsic) and endogenous (intrinsic) erosive factors can be differentiated. Extrinsic factors include erosions resulting from diet or habits, such as a high consumption of acidic soft drinks, sports drinks, or regular alcohol consumption [5,6]. Intrinsic factors, such as diseases that provoke reflux, can also cause erosions. Dry mouth can also lead to increased acid-related defects due to reduced salivary flow and the associated reduced buffering capacity [5,7,8].

The prevalence for erosion-related tooth surface loss in Germany has been estimated as 45% for children, 30% for adolescents [9], and 20% to 45% for adults [10]. Tooth surface loss can lead to increased tooth sensitivity, cause an impaired aesthetic appearance, or foster the onset of functional problems [11]. In the case of extensive tooth surface loss with affected supporting zones of the dentition or extension far into the dentin, it may be necessary to rehabilitate the affected teeth by prosthetic means to restore and secure the horizontal and vertical dimensions of occlusion. In addition to indirect restorations fabricated from metal and ceramics, computer-aided design/computer-aided manufacturing (CAD/CAM) resin composites may be used for this purpose. CAD/CAM resin composites are based on a polymeric matrix composed of different kinds of dimethacrylates (DMA) that are supplemented with high volumes of inorganic fillers (mostly glasses). The fillers differ in shape, size, and composition [12]. In comparison to direct resin-based composites used for fillings, CAD/CAM resin composite blocks or discs are produced under optimized and standardised industrial polymerisation conditions. As a result, the degree of polymerisation and, therefore, the mechanical performance of CAD/CAM resin composites are better than those of direct resin-based composites [13]. Due to their lower brittleness compared to ceramics, these materials can be designed with lower minimum layer thicknesses and thinner margins, which requires less invasive preparation of the abutment teeth [14]. In addition, less chipping and intraoral reparability are advantageous properties of CAD/CAM resin composites [15,16]. Furthermore, in comparison to ceramics, CAD/CAM resin composites are beneficial in terms of their hardness, strength, and elastic moduli, which are comparable to tooth structure [17,18]. This leads to less wear of antagonist enamel [13,15,17].

Despite the favourable material properties of CAD/CAM resin composites, it is unclear whether the performance of these materials in dental restorations frequently exposed to acids will be adequate over longer periods. Acids may affect the roughness, abrasion resistance, and hardness of resin-based composites in vitro [19,20,21]. A case series in patients with eroded teeth showed that the shape, surface finish, and marginal quality of direct resin-based composite restorations deteriorate relevantly over a five-year period, and discolouration at the margins may also occur [22].

With regard to this aspect, the acid resistance declared as chemical solubility has, to date, only sporadically been investigated for distinct materials such as ceramics. The process included immersion in 4% acetic acid heated to 80 °C for a period of 16 h and subsequent determination of the loss of mass [23]. However, no standardised test methods for evaluating the acid resistance of CAD/CAM resin composites are currently available [24]. In contrast to ceramics, CAD/CAM resin composites are generally not tested by manufacturers within the scope of approval. A simple transfer of the regulated test specification for ceramic materials is not expedient, since different mechanisms of action apply to resin-based materials and other acids. A recently published study highlighted that the surface quality of CAD/CAM resin composites is less susceptible to dietary solvents than is the quality of direct resin-based composites. At the same time, the study recommends further investigations and longer storage periods to adequately assess the material-specific long-term properties of CAD/CAM resin composites [25]. Research on the leaching behaviour of dental resin-based composites by approximately pH-neutral liquids such as water or artificial saliva suggests that the material properties (strength, fracture behaviour, hardness) and the surface properties of CAD/CAM resin composites, which are decisive for microbiology, are affected by exposure to acids [26,27].

By using selected CAD/CAM resin composites, this study evaluated their resistance against different acidic solutions (tonic water, acetic acid, hydrochloric acid) and control medium (demineralized water) in terms of surface roughness (Sa) and Vickers hardness (HV). Based on current knowledge, the null hypothesis of our study was that exposure to acid solution does not significantly affect the near-surface properties, such as the hardness and roughness, of CAD/CAM resin composites.

## 2. Materials and Methods

### 2.1. Specimen Preparation

Five materials were investigated for the in vitro analysis of the acid resistance of CAD/CAM resin composites (Table 1).

For the fabrication of the specimens, the corresponding CAD/CAM resin composite blocks were cut by using a precision saw (IsoMet4000, Buehler Ltd., Lake Bluff, IL, USA) under water cooling. Then, the specimens were subjected to a constant grinding and polishing regime by using a semiautomatic polishing device (Pedemin-2/DAV-5, Struers GmbH, Willich, Germany). Sandpaper discs up to P500 and grinding discs with a diamond suspension down to a particle size of 0.04 µm were used. After the polishing process, the slices had dimensions of 13 ± 1 × 14.5 ± 0.5 × 2.0 ± 0.2 mm^3^. This resulted in a ratio of sample surface to media volume of 4–5 mm^2^ mL^−1^. Finally, the specimens were cleaned with demineralized water, dried for 17 h at 40 ± 1 °C, and then stored in a desiccator. Four different media were used to stress the CAD/CAM test specimens (Table 2), which were selected in order to cover a wide range of frequently present acid-related erosive factors with a high erosive potential (see Section 1). For each specimen, 100 mL of the medium was initially pretempered in 250 mL screw-top vials at 40 ± 1 °C (the lower temperature was selected for imaging clinically relevant strains) in the drying cabinet. After stress loading for 232 h (t_1_), the specimens were removed from the medium, cleaned with demineralized water, and dried with subsequent storage in a desiccator to prevent an undesirable influence on the results (especially mass, dimensions, microhardness) due to, e.g., impurities or pure swelling processes, as they occur in dental resin composites, and to ensure comparability of the test [28]. Constant stressing was ensured by monitoring of the pH value and the conductivity (σ) in each medium before and after insertion of the respective specimen. A data logger (ALMEMO^®^ 2590 A, Ahlborn Mess- und Regelungstechnik GmbH, Holzkirchen, Germany), which was connected with a pH sensor (DULCOTEST PHER 112 SE, ProMinent, Heidelberg, Germany), a σ-sensor (FYA641LFP1, Ahlborn Mess- und Regelungstechnik GmbH, Holzkirchen, Germany), and a temperature sensor (R2E4, Ahlborn Mess- und Regelungstechnik GmbH, Holzkirchen, Germany) were used to analyse the media before and after loading.

### 2.2. Roughness Parameters

The measurements to determine the surface roughness prior to (t_0_) and after (t_1_) loading were performed by confocal laser-scanning microscopy (CLSM) (VK-X1000/1050, Keyence, Osaka, Japan). For each specimen and loading cycle, two measurements were performed by using a 50× objective (CF IC EPI Plan 50×; N = 0.8; WD = 0.54 mm) on eight areas on the entire surface of each specimen to minimize the influence of local discontinuities on the surface analysis.

The recording and calculation of surface roughness was conducted with manufacturer software (VK Viewer 1.1.2.174 and MultiFileAnalyzer 2.1.3.89, both from Keyence, Osaka, Japan). A consistent correction was carried out for all surface scans. The form of the specimen surface was filtered out with a cut-off of 0.2 mm (F-filter). An S-filter of 0.5 µm was applied to analyse surface details based on an S-F-surface. In both approaches, a Gaussian filter was used, and an end effect correction was performed. To ensure unaltered surface characteristics of the Sh samples through filtering, an L-filter was deliberately omitted. For the subsequent determination of the surface roughness, each scan of the S-F-surface was divided into four areas (each 100 µm × 100 µm). The arithmetical mean height (Sa) for each area was determined for each material group, media, and loading cycle [29]. A positive ΔSa represents an increase, and a negative ΔSa represents a decrease in surface roughness.

### 2.3. Determination of Vickers Hardness

Vickers hardness (HV) was determined prior to (t_0_) and after (t_1_) loading by using a microindentation tester (MHT-4 Anton Paar, Graz, Austria) with a pyramid-shaped diamond indenter. Each specimen was placed on the examination plate with the tested surface facing the indenter. On the surface of each specimen, three indentations with a testing force of 200 p = 1.961 N, a testing time of 12 s, and a testing speed of 35 p s^−1^ = 0.343 N s^−1^ were produced. Sufficient distance of the indentations from each other and from the edge of the specimen was verified [30]. To determine HV, two-point measurements via CLSM were applied to the indentation diagonals. HV was calculated according to Formula (1):

HV = 0.1891 × F × d^−^^2^ (1)

F = testing force (N); d = diagonal length of indentation (mm).

### 2.4. Statistical Analysis

Statistical analysis was performed (SPSS 25, SPSS Inc., IBM, Chicago, IL, USA), and normal distribution was determined by using the Kolmogorov–Smirnov and Shapiro–Wilk tests. Based on this evaluation, the data were analysed with paired t-test or Wilcoxon signed-rank tests to compare the material-specific properties in the various media. Surface roughness and HV were compared by using two-way ANOVA and multiple comparison analysis post-hoc test under Bonferroni correction. Statistical analysis was conducted at a significance level of α = 0.05.

### 2.5. Further Analysis to Investigate the Damage Mechanisms

Scanning electron microscopy (SEM) with energy dispersive X-ray spectroscopy (EDS) and micro X-ray computer tomography (µXCT) was used to qualitatively investigate changes in the microstructure of selected stressed materials that showed the strongest altered surface characteristics. As only a few samples showed changes, extensive investigations were carried out on an exemplary basis.

For the SEM–EDS measurements, two specimens of the selected material—one stressed in M_H2O_ and the other stressed in M_HCl_—were sectioned and embedded in resin with the cross section facing upwards and were polished and sputtered with 10 nm Cr to prevent charging effects. The cross-section to the three surfaces that were previously in contact with the storage media was investigated. Measurements were performed with 5 kV (SEM images) and 15 kV (EDS mappings) on an Ultra 55 SEM (Carl Zeiss Microscopy GmbH, Jena, Germany) with an SE2 detector. EDS-Mappings were performed to investigate differences in elemental distribution (O, Al, Si, Ti, Cr, Ba, C) from the outer to the inner areas.

A directional X-ray tube “FXE 225.99” (200 kV, 150 µA, focal spot 0.6 µm, tungsten target) from YXLON International GmbH and a 2D-detector “1621 N” (2048 × 2048 pixels, CsI, pitch 200 µm) from PerkinElmer Inc. were used to analyse the cylindrical samples (Ø 2 mm) of selected material Ce before and after the stress caused by M_HCl_. The edge length of the voxels was 2.8^3^ µm^3^. The raw data were cut, oriented, and calibrated with ImageJ (National Institutes of Health, 1.51d, Bethesda, MD, USA) in accordance with Koenig (2020) [31].

## 3. Results

### 3.1. Surface Roughness

The evaluation of the S-F-surface (filtered surface, see Section 2.2) for all specimens revealed the following gradation for the roughness parameter Sa before exposure to the storing media (t_0_): Sh > La > Gr > Br > Ce (*p* ≤ 0.031); only Br did not significantly differ from Gr and Ce. The highest Sa value was recorded for Sh with 0.036 µm, and the lowest was recorded for Ce with 0.008 µm (Supplementary Table S1).

After exposure to the various media (t_1_), the gradation of mean Sa was (t_1_): Sh > Gr > Ce > La > Br (*p* ≤ 0.035), with no significant differences between Br and La and a significant difference between Ce and Sh. The Sa value of Sh was significantly higher than those of all other CAD/CAM resin composites (*p* < 0.001), with a maximum value of 0.038 µm after storage in M_AcOH_. With respect to the media, the roughness gradation after immersion (t_1_) was as follows: M_AcOH_ > M_HCl_ > M_H2O_ > M_TW_ (*p* ≤ 0.003), while M_H2O_ and M_TW_ did not significantly differ from each other.

**Figure 1:**
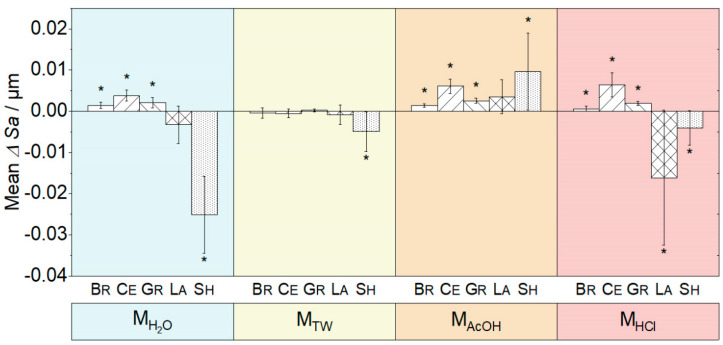
Changes in the mean and standard deviation of the surface roughness (Sa) (t_1_−t_0_) due to immersion in the different media M_H2O_ (demineralized water), M_TW_ (tonic water), M_AcOH_ (acetic acid) and M_HCl_ (hydrochloric acid) (* indicates *p* < 0.05).

For the mean changes in Sa values ΔSa (t_1_−t_0_), a similar trend was observed (Figure 1). The smallest changes were observed with M_TW_. After exposure to M_AcOH_ (with the exception of La) and M_HCl_, all CAD/CAM resin composites showed a significant change in Sa (*p* ≤ 0.044). The maximum increase in Sa (ΔSa = +0.010 µm) was observed with M_AcOH_ for Sh, followed by Ce in M_HCl_, M_AcOH_, and M_H2O_. In addition, the maximum decrease in Sa was determined for Sh (ΔSa = −0.025 µm) after storage in M_H2O_.

**Figure 2:**
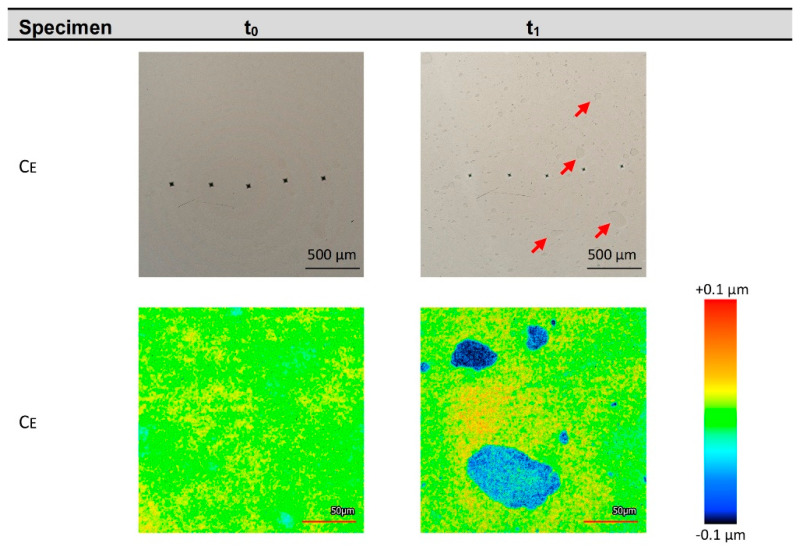
Surface of the Ce specimen before (t_0_) and after (t_1_) immersion in M_HCl_. Above: Ce overview images of the same specimen location (the five pyramidal indentations of the hardness determination were used as orientation). Multiple darker spots manifested on the Ce surface (see red arrows at top right) due to immersion in M_HCl_. Surface rendering revealed that the spots are depressions. Below: height-scaled false colours.

In comparison with the other CAD/CAM resin composites, only the surface of Sh was very heterogeneous, with more or less semispherical hills both before and after loading in the storing media (Supplementary Figure S1). The change here was not immediately apparent by examining the surface images. In contrast, Ce showed a relatively homogeneous surface before immersion in M_HCl_. After stressing, clear changes appeared in the form of heterogeneously distributed spots of greater depth (Figure 2).

### 3.2. Vickers Hardness

The Vickers hardness of the CAD/CAM resin composites revealed the following gradation before exposure to storing media (t_0_): Gr > La > Br > Ce > Sh (*p* < 0.001), while Br, Ce, and Sh did not significantly differ. The largest value of HV 161.5 was observed for Gr and the smallest for Sh with HV 66.8 (Supplementary Table S2).

The HV results after exposure to the different media were ranked as follows (t_1_): Gr > La > Ce > Br > Sh (*p* ≤ 0.001); Br did not significantly differ from Ce and Sh. The HV values after immersion (t_1_) ranged from 66.5 for Sh after M_TW_ to 161.6 for Gr after M_AcOH_ exposure. The following trend in mean HV was assessed after exposure to the media (t_1_): M_AcOH_ > M_H2O_ > M_TW_ > M_HCl_ (*p* ≤ 0.007), with only the value after M_HCl_ HV being significantly smaller than those after M_AcOH_ and M_H2O_.

**Figure 3:**
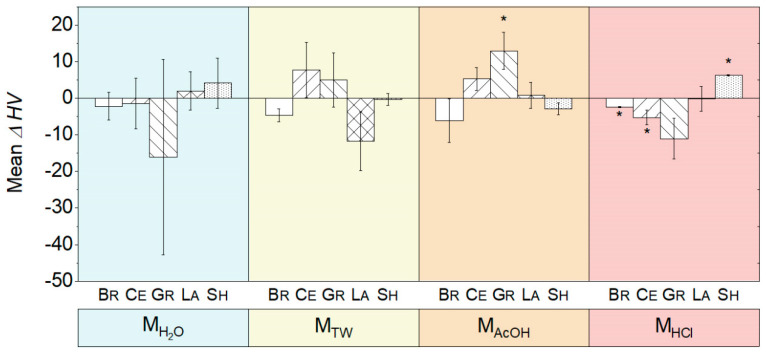
Changes in the mean and standard deviation of the Vickers hardness (HV) (t_1_−t_0_) due to immersion in the different media M_H2O_ (demineralized water), M_TW_ (tonic water), M_AcOH_ (acetic acid), and M_HCl_ (hydrochloric acid) (* indicates *p* < 0.05).

The mean changes in HV values ΔHV (t_1_−t_0_) presented no significant changes after storage in M_H2O_ and M_TW_ (Figure 3). Through exposure to M_AcOH_, Gr experienced the maximum increase in ΔHV of +12.9 (*p* ≤ 0.047). The highest changes occurred after exposure to M_HCl_: Br (ΔHV = −2.4) and Ce (ΔHV = −5.2; maximum decrease), which was significant (*p* ≤ 0.044), whereas HV for Sh increased by +6.3 (*p* < 0.001).

### 3.3. Damage Mechanisms

Only the Ce material presented a significant reduction in HV in combination with increased Sa and a clear spatially resolved change in the surface after immersion in M_HCl_ (Figure 2). Therefore, the microstructure of this material was selected for detailed investigations by SEM–EDS and µXCT after storage in water (M_H2O_) and hydrochloric acid (M_HCl_).

**Figure 4:**
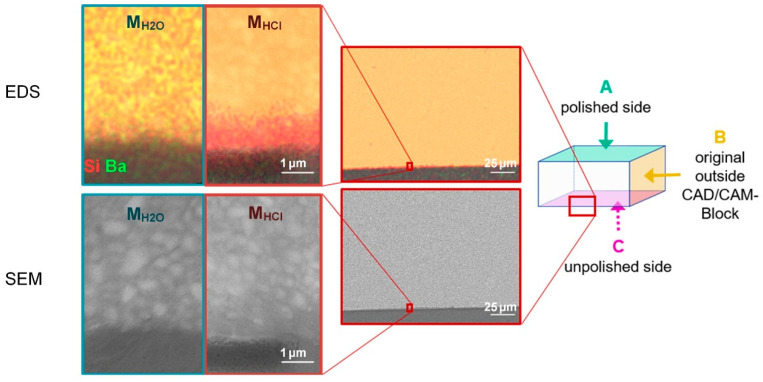
Visualization of the investigated Ce cross section. SEM images (bottom) and EDS mapping (top; Si, Ba) at the edge of the unpolished side C. Images of samples stressed in M_HCl_ with a red frame (and in M_H2O_ with a blue frame for comparison). Note that yellow areas result from an overlay of red Si and green Ba.

SEM images of Ce after exposure to M_H2O_ and M_HCl_ presented the morphology of filler particles embedded in a polymeric matrix typical for CAD/CAM resin composites (Figure 4). SEM–EDS mappings of the edges to side A and side B revealed no differences in elemental distribution and morphology between Ce stressed in M_H2O_ and M_HCl_.

However, the cross sections at the edge of the unpolished side **C** showed clear differences between Ce stressed in M_H2O_ and M_HCl_ in terms of morphology and elemental distribution (Figure 4). While SEM images of different edges stressed in M_H2O_ revealed no differences, Ce stressed in M_HCl_ had a zone of approximately 2 µm distance from the edge with particles that were less radiopaque than the more inward particles. The EDS mappings of the area confirmed the absence of titanium, aluminium, and barium in this layer, while the distribution of the other elements remained the same.

**Figure 5:**
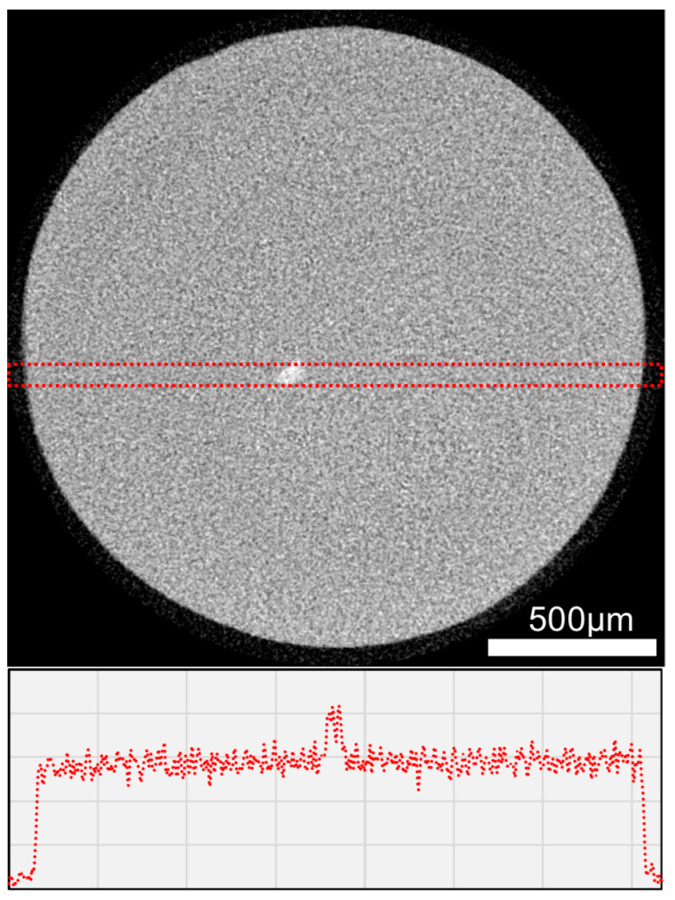
Cross section (**top**) from Ce (cylindrical sample) based on µXCT with grey value distribution (**below**) in the region of interest (red dotted rectangle) after storage in hydrochloric acid.

The µXCT investigations of Ce specimens before and after storage in hydrochloric acid have shown, taking into account the resolution of 2.8 µm, no changes in the grey value distribution and no material erosion at the surface (Figure 5).

## 4. Discussion

The null hypothesis of the present study, which posits that exposure to acidic media does not significantly affect the near-surface properties such as the hardness and roughness of CAD/CAM resin composites, can be partly rejected. All five CAD/CAM resin composites examined in this study showed significant changes in either roughness (Sa) or Vickers hardness (HV) after exposure to at least one of the media: demineralized water (M_H2O_), tonic water (M_TW_), acetic acid (M_AcOH_), and hydrochloric acid (M_HCl_).

The main limitation of this in vitro study in comparison to clinical practice is the reduction in the number of simultaneously interacting types of stress. It is conceivable that in reality, the mechanical stress forms, e.g., abrasion, increase the progress of the damage caused by the acids.

Despite their statistical significance, the magnitude of the changes in roughness with max. ΔSa = −0.025 µm (Sh in M_H2O_) and in Vickers hardness with max. 12.9 in ΔHV (Gr in M_AcOH_) were relatively small (Figure 3) in relation to the storage time (232 h). The change in Sa was approximately one-tenth of the roughness differences that, according to Jones et al. (2004), can be detected by the human tongue [32]. The change in HV (9%) was approximately three-quarters of the maximum percentage change observed by Sagsoz et al. (2019) after one week of ageing CAD/CAM ceramics in lactic or citric acid [33]. The intermaterial differences (max. 59%) far exceed the changes caused by acidic media. At the same time, even though the storage time was longer within the present study (232 h), the magnitudes of changes in roughness are similar to the maximum changes found by Munusamy et al. (2020); they observed a change in roughness Ra of 0.022 µm between La aged for 168 h in lactic acid (Ra = 0.142 µm) and the control air (Ra = 0.120 µm) [25].

With regard to roughness, the yielded maximum absolute value in the present investigation of Sa = 0.038 µm (Sh in M_AcOH_) was due to a change of 0.010 µm (ΔSa) compared to Sa = 0.028 µm in the sample before immersion. This was lower than the surface roughness limit of Ra = 0.2 µm, which is crucial for bacterial adhesion [34,35,36,37]. Furthermore, a lower roughness no longer reduces bacterial retention on dental materials [35]. In addition, CAD/CAM materials with surface finishes for clinical purposes often present an even greater surface roughness [38], thus accounting for the precise labside polishing process performed in this study. The surface roughness values of high gloss polishing of CAD/CAM resin composites described in other publications with a labside approach [38,39] are comparable to the surface roughness Sa values determined in the present study. However, regulations are needed for a definite testing and evaluation concept for the acid resistance of dental CAD/CAM resin composites and resins.

Although the changes in Sa and HV were small, the differences induced through immersion in the media varied in number and intensity. When considering the effects of the different media on the CAD/CAM resin composites, it was noticeable that M_TW_ had a lower effect on the roughness (Sa) than M_H2O_, despite the lower pH of M_TW_. This observation is in contrast to the results of Munusamy et al. (2020) [25], who noticed significantly increased roughness of Ce, La, and Sh after 168 h immersion in citric acid (with a similar pH value to the tonic water used herein, which also contains citric acid) at 37 °C compared to demineralized water [25]. A possible explanation might be the prolonged time (232 h) and higher temperature (40 °C) applied in the current study. Munusamy et al. (2020) suggested that demineralized water might exert significant effects on roughness after longer conditioning times [25]. In the same way, Rosentritt et al. [39] were able to demonstrate water saturation after 90 to 180 days and effects in the resin by thermoanalytical studies. These effects can indicate ageing by water with increasing storage time [39]. Other factors might be explained by the lower ionic strength of demineralized water that enhances leaching, swelling, and causes the presence of additional ingredients in the medium M_TW_ (compared to the pure citric acid in [25]), which may reduce the effect. In terms of Vickers hardness, no significant differences between M_H2O_ and M_TW_ were observed.

The maximum values of Sa and the maximum changes ΔSa were detected for Sh. The authors attribute this to the high heterogeneity of the Sh surface even after a strict polishing protocol (Supplementary Figure S1). Compared to all other investigated CAD/CAM resin composites, filler particles of Sh are predominantly spherical in nature [12]. This entails a minimal ratio between surface and volume. As a consequence, the bonding area between the filler and resin matrix is minimal compared to the other CAD/CAM resin composites. Presumably, the fillers of Sh have a poorer bond to the resin matrix and can easily dissolve during abrasion. The heterogeneity of the Sh surfaces is also reflected by the high standard deviations.

In addition to the already discussed heterogeneity of Sh, the decreases in surface roughness Sa observed for Sh (M_H2O_, M_TW_, M_HCl_) and La (M_HCl_) in the respective media could also be explained by swelling. In the present study, this effect was limited due to the drying procedure before stressing the specimen: however, in general, it is well known for dental composites [28,40,41]. It is easy to imagine that when a relatively soft polymer matrix swells between rigid filler particles, the polymer matrix lifts due to the incorporation of solvent. Thus, the height differences would be levelled, and the surface roughness would be reduced. This effect was highest for M_H2O_ (Sh), which supports the interpretation. The increased Vickers hardness for Sh in M_HCl_ (ΔHV = 6.3) and for Gr in M_AcOH_ (ΔHV = 12.9) may also be explained by swelling in combination with postcuring [42]. Moraes et al. (2008) [42] explain the increase in Knoop hardness after water storage with increased monomer conversion or post-curing due to a crosslinking within the polymer matrix. The influences of large-scale local discontinuities in several CAD/CAM resin composites might also serve as a potential explanation for these unexpected observations [12].

For Ce, the most pronounced surface changes were observed in M_H2O_, M_AcOH_, and M_HCl_. This was reflected by a strongly increased surface roughness parameter Sa (Figure 3). In contrast to the other CAD/CAM resin composites, this was already obvious with optical microscope images even without surface roughness analysis (Figure 2). Furthermore, the most severe reduction in Vickers hardness HV was present for Ce in M_HCl_. In general, degradation of CAD/CAM resin composites can be attributed to the direct attack towards the polymeric matrix, the leaching of filler particles, or the weakening of the bonding between fillers and matrix—addressing the silanisation [43]. Various examples exist for the hydrolysis of ester bonds that are present in the polymeric matrix of CAD/CAM resin composites [25,44,45]. Hence, it seems reasonable that at least some of the induced changes in Ce were due to degradation of the polymer matrix. The filler particles of some CAD/CAM resin composites, such as Ce, contain barium [17]. Söderholm et al. (2000) observed that barium-containing filler particles are susceptible to leaching [46].

Taking into account the resolution of 2.8 µm, the µXCT investigations showed that the grey value distribution in Ce and the diameter of the specimens did not change after exposure to hydrochloric acid. Despite the effects on the surface roughness and Vickers hardness of Ce, no deep damage was detected by µXCT. This is in accordance with the SEM–EDS results observed on the cross sections of Ce in M_H2O_ and M_HCl_ (Figure 4). Only after stressing in M_HCl_ did Ce show a very narrow leached zone of approximately 2 µm (below the detection limit of µXCT). Therefore, only the surface, not the internal structure of Ce, was changed by the acids. The elements leached were barium, titanium, and aluminium. As these are components of the filler particles in dental resin composites [12,47,48], it is reasonable to assume that the changes in surface roughness and Vickers hardness of Ce after M_HCl_ exposure are attributable to filler leaching. Moreover, filler leaching was only associated with the surface. The polymer matrix consists mainly of lightweight elements such as carbon or hydrogen. As EDS measurements are less sensitive to hydrogen, some changes in the volume of the polymer matrix might remain undetected.

A possible explanation for the fact that only the unpolished side seemed to be attacked by M_HCl_ is that rough surfaces have high surface energy with scratches or holes. This is where dissolution processes of minerals and glasses are easily initiated, since less energy is needed to break the remaining bonds [49]. The increased surface area also accelerates the process. Buchwalter et al. (1982) reported that, in general, a higher surface roughness of simple silicate glasses increases their dissolution [50].

Taking our results and the limitations of the study into account, it can be assumed that the polishing process can increase the acid resistance for some CAD/CAM resin composites, such as Ce. Although the present study design only investigated roughness as an effect of acid attack on CAD/CAM resin composites, there are indications of an increased susceptibility to acid exposure due to initially rougher surfaces. This should be investigated in further studies.

## 5. Conclusions

The resistance of five CAD/CAM resin composites against three relevant acidic media (tonic water, acetic acid, hydrochloric acid) and demineralized water were investigated by means of the surface roughness parameter Sa and the Vickers hardness. Within the limitations of this in vitro study, the null hypothesis that near-surface properties will not change significantly by acid solutions was partly rejected. The investigations revealed the following:

(1) All of the investigated CAD/CAM resin composites were susceptible to at least one of the applied media.
(2) The changes in surface roughness (max. ΔSa = −0.025 µm) and Vickers hardness (max. ΔHV = 12.9) induced through the different media were small with regard to storage time, literature values, and clinically relevant thresholds [25,32,33,35].
(3) The greatest differences were observed for Cerasmart after storage in hydrochloric acid. Further investigations via SEM–EDS and µXCT revealed leached fillers with reduced quantities of barium, aluminium, and titanium that were present in a 2 µm surface region of the rough surfaces. The comparison between polished and unpolished surfaces suggested that acid resistance may increase with lower surface roughness.

In conclusion, the CAD/CAM resin composites showed sufficient resistance against different acidic media and demineralized water. For clinical usage, CAD/CAM resin composites can be recommended for use under erosive conditions. Nonetheless, regulations are needed for a definitive testing and evaluation concept for the acid resistance of CAD/CAM resin composites. Further investigations that focus on the possible influence of acids on the polymeric matrix and the effect of initial roughness on filler leaching behaviour remain interesting for future investigations.

## Figures and Tables

**Table 1:** CAD/CAM composites used in this study and composition of the inorganic fillers [12,17].

Material	Abbreviation	Manufacturer	Lot No.	Inorganic Filler
BRILLIANT Crios	Br	Colténe, Altstätten, Switzerland	H96172 (block) IO3077 (package)	barium glass <1.0 µm; amorphous silica <20 nm
CERASMART	Ce	GC, Bad Homburg, Germany	1710041	silica and barium glass nanoparticles
Grandio blocs	Gr	VOCO, Cuxhaven, Germany	1831584	nanohybrid fillers
Lava Ultimate	La	3M, St. Paul, MN, USA	N401476	silica nanomers 20 nm; zirconia nanomers 4–11 nm; zirconia-silica nanoclusters 0.6–10 µm
SHOFU Block HC	Sh	Shofo, Kyoto, Japan	0818225	silica-based glass and silica

**Table 2:** Liquids to which the test specimens were exposed.

Media	Abbreviation	Manufacturer	pH Value	Concentration (mol L^−1^ )
Demineralized water	M_H2O_	-	-	-
Tonic water	M_TW_	Schweppes Deutschland, Kreuztal, Germany	2.59	Degassed
Acetic acid	M_AcOH_	Carl Roth, Karlsruhe, Germany	2.48	0.94
Hydrochloric acid	M_HCl_	Carl Roth, Karlsruhe	1.68	0.03

## Data Availability

Not applicable.

## Supplementary Materials

The following supporting information can be downloaded at: https://www.mdpi.com/article/10.3390/biomedicines10061383/s1, Figure S1: Surface of the CAD/CAM resin composite specimen before (t_0_) immersion in the different media; Table S1: Media-specific surface roughness (Sa) of the different specimens: BRILLIANT Crios (Br), CERASMART (Ce), Grandio blocs (Gr), Lava Ultimate (La), SHOFU Block HC (Sh).; Table S2: Media-specific Vickers hardness (HV) of the different specimens.
