# Supplementary material for: Acid Resistance of CAD/CAM Resin Composites
Source: Biomedicines. 2022 Jun 11;10(6):1383. doi: 10.3390/biomedicines10061383 (PMC9220078; doi:10.3390/biomedicines10061383)
Supplement: Supplementary file 1 [file biomedicines-10-01383-s001.zip › biomedicines-1737471-supplementary.pdf]

Supplementary Materials

Table S1. Media-specific surface roughness (Sa in µm) of the different specimens: BRILLIANT Crios (Br), CERASMART (Ce), Grandio blocs (Gr), Lava Ultimate (La), SHOFU Block HC (Sh).

Specimen	Media	Sa (Mean t_0)	Sa (Mean t_1)	Δ_{t1-t0}	Standard deviation	*p* Value
Br	M_{H2O}	0.012	0.014	0.002	0.001	**<0.001**
	M_{TW}	0.011	0.011	−0.001	0.001	0.157
	M_{AcOH}	0.011	0.012	0.002	0.001	**0.010**
	M_{HCl}	0.010	0.011	0.001	0.001	**0.023**
Ce	M_{H2O}	0.009	0.015	0.004	0.001	**<0.001**
	M_{TW}	0.008	0.014	−0.001	0.001	0.180
	M_{AcOH}	0.010	0.009	0.006	0.002	**0.012**
	M_{HCl}	0.012	0.016	0.007	0.003	**0.011**
Gr	M_{H2O}	0.014	0.016	0.002	0.001	**0.004**
	M_{TW}	0.014	0.014	0.000	0.000	0.351
	M_{AcOH}	0.014	0.016	0.003	0.001	**0.010**
	M_{HCl}	0.013	0.015	0.002	0.000	**0.007**
La	M_{H2O}	0.017	0.014	−0.003	0.004	0.088
	M_{TW}	0.012	0.011	−0.001	0.003	0.394
	M_{AcOH}	0.010	0.014	0.004	0.004	0.058
	M_{HCl}	0.030	0.014	−0.016	0.017	**0.018**
Sh	M_{H2O}	0.036	0.011	−0.025	0.009	**0.012**
	M_{TW}	0.023	0.018	−0.005	0.005	**0.020**
	M_{AcOH}	0.028	0.038	0.010	0.009	**0.022**
	M_{HCl}	0.030	0.026	−0.004	0.004	**0.025**

**Specimen**	**t_0**		
Br	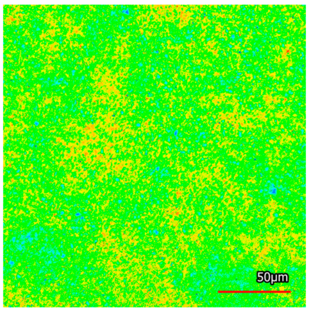	La	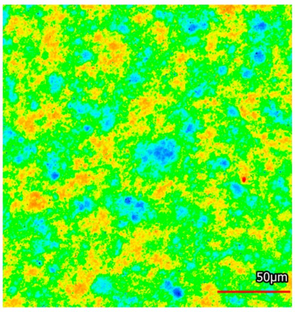
Ce	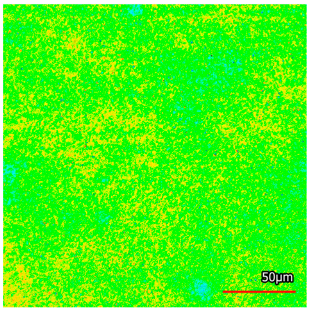	Sh	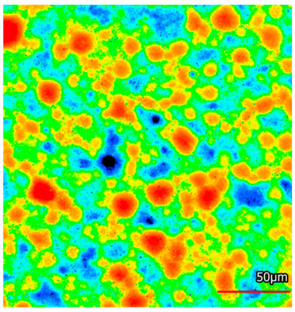
Gr	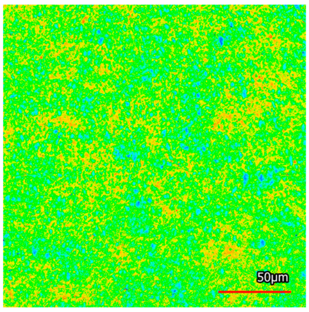		+0.1 µm 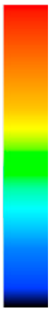 −0.1 µm

Figure S1. Surface of the CAD/CAM resin composite specimen before (t_0) immersion in the different media. Representative surface renderings are shown in height-scaled false colours. A pronounced heterogeneity can be observed in the surfaces of Sh compared to the other CAD/CAM resin composites: BRILLIANT Crios (Br), CERASMART (Ce), Grandio blocs (Gr), Lava Ultimate (La), SHOFU Block HC (Sh).

Table S2. Media-specific Vickers hardness (HV) of the different specimens.

Specimen	Media	HV (Mean t_0)	HV (Mean t_1)	Δ_{t1-t0}	Standard deviation	*p* Value
BR	M_{H2O}	79.3	77.1	-2.2	3.9	0.434
	M_{TW}	82.0	77.3	-4.7	1.8	0.109
	M_{AcOH}	84.6	78.5	-6.1	6.0	0.220
	M_{HCl}	81.5	79.1	-2.4	0.2	**0.003**
CE	M_{H2O}	80.5	79.0	-1.5	6.9	0.750
	M_{TW}	84.2	91.9	7.7	7.6	0.219
	M_{AcOH}	69.7	74.9	5.3	3.1	0.109
	M_{HCl}	77.6	72.4	-5.2	2.0	**0.044**
GR	M_{H2O}	161.5	145.4	-16.1	26.8	0.408
	M_{TW}	147.5	152.5	5.0	7.4	0.362
	M_{AcOH}	148.7	161.6	12.9	5.1	**0.047**
	M_{HCl}	157.6	146.6	-11.0	5.6	0.075
LA	M_{H2O}	114.5	116.4	2.0	5.2	0.582
	M_{TW}	118.2	106.5	-11.7	8.1	0.109
	M_{AcOH}	113.3	114.0	0.7	3.5	0.751
	M_{HCl}	112.0	111.9	-0.1	3.4	0.958
SH	M_{H2O}	78.9	83.0	4.1	6.9	0.285
	M_{TW}	66.8	66.5	-0.4	1.6	0.742
	M_{AcOH}	78.7	75.9	-2.8	1.6	0.095
	M_{HCl}	70.5	76.8	6.3	0.2	**< 0.001**
